# Circadian Sensitivity of Noise Trauma-Induced Hearing Loss and Tinnitus in Mongolian Gerbils

**DOI:** 10.3389/fnins.2022.830703

**Published:** 2022-06-03

**Authors:** Jannik Grimm, Holger Schulze, Konstantin Tziridis

**Affiliations:** Experimental Otolaryngology, University of Erlangen-Nuremberg, Erlangen, Germany

**Keywords:** rodents, tinnitus, chronobiology, acoustic trauma, gap prepulse inhibition of the acoustic startle response, auditory brainstem response audiometry

## Abstract

Noise-induced hearing loss (HL) has a circadian component: In nocturnal mice, hearing thresholds (HT) have a significantly stronger effect to acoustic trauma when induced during the night compared to rather mild effects on hearing when induced during daytime. Here, we investigate whether such effects are also present in diurnal Mongolian gerbils and determined whether trauma-induced HL correlated with the development of a tinnitus percept in these animals. In particular, we investigated the effects of acoustic trauma (2 kHz, 115 dB SPL, 75 min) on HT and tinnitus development in 34 male gerbils exposed either at 9 AM, 1 PM, 5 PM, or 12 PM. HT was measured by acoustic brainstem response audiometry at defined times 1 day before and 1 week after the trauma. Possible tinnitus percepts were assessed behaviorally by the gap prepulse inhibition of the acoustic startle response at defined times 1 day before and 1 week after the trauma. We found daytime-dependent changes due to trauma in mean HT in a frequency-dependent manner comparable to the results in mice, but the results temporally shifted according to respective activity profiles. Additionally, we found linear correlations of these threshold changes with the strength of the tinnitus percept, with the most prominent correlations in the 5 PM trauma group. Taken together, circadian sensitivity of the HT to noise trauma can also be found in gerbils, and tinnitus strength correlates most strongly with HL only when the trauma is applied at the most sensitive times, which seem to be the evening.

## Introduction

Circadian rhythms substantially affect the behavior and physiology of animals (Konopka and Benzer, 1971; Gachon et al., 2004; Richards and Gumz, 2013). Underlying mechanisms are centrally controlled by the suprachiasmatic nucleus in the hypothalamus (Moore and Eichler, 1972) and synchronized to light emission received *via* the eyes. In addition, sound can entrain circadian rhythms by modulating melatonin production or body temperature (Goel, 2005), and circadian clocks were found in the cochlea and the inferior colliculus (Meltser et al., 2014; Park et al., 2016). This is based on variable mRNA expression and protein production, possibly affecting neuronal processing within the auditory system over the course of the day (Park et al., 2016).

Effects of such circadian clocks on the auditory system are evident in auditory-related behavior such as the startle response to a loud sound. In mice, a nocturnal species (Ripperger et al., 2011), it has been shown that the amplitude of the acoustic startle response (ASR) is larger during daytime (their resting or inactive phase) compared to the night (active phase) (Meltser et al., 2014). In other nocturnal species such as rats (Stephan, 1983) or humans as a diurnal species (Miller and Gronfier, 2006), this effect has been shown to be reversed (Frankland and Ralph, 1995). Interestingly, such internal clocks in mice not only affect auditory processing but also modulate the effects of noise trauma on the hearing thresholds (HT). Then, 2 weeks after acoustic trauma, the trauma-elevated thresholds completely recovered in CBA/Ca/Sca male mice, which received trauma (6–12 kHz bandpass noise, 100 dB SPL, 60 min) during the inactive phase. On the other hand, animals that received trauma during the active phase showed a permanent high-frequency hearing loss (HL) (Meltser et al., 2014). Furthermore, the brain-derived neurotrophic factor (BDNF) mRNA and protein expression-related responses of the internal clocks, e.g., in the cochlea and inferior colliculus in the latter animals were significantly reduced, indicating a lack of BDNF-related protective response ability after active phase trauma (Fontana et al., 2019). Nevertheless, the results can vary dependent on the species or even strain used for the animal experiments (Meltser et al., 2014; Harrison, 2019; Sheppard et al., 2019) and might not be usable for a direct comparison with human physiology. We here attempted to reduce at least one of the constrains with rodents, namely, being mostly nocturnal animals, using a diurnal rodent species that in our view showed to be a good model for human HL and tinnitus development after an acoustic trauma.

In this study, we investigated whether an acoustic trauma applied at different times of the day in diurnal male Mongolian gerbils (Roper, 1976) leads to variable changes in HT. If so, we were furthermore interested in the question, of whether different trauma effects on HT also lead to a different rate and/or strength of tinnitus development in these animals. Our hypotheses were, first, that gerbils show a higher sensitivity to acoustic trauma during their active phase and, second, that tinnitus development is different at different times based on the differences in HL. The fact that tinnitus severity and the amount of hearing loss are correlated in humans is well described [e.g., (Searchfield et al., 2007)], but tinnitus strength/loudness and severity are not necessarily identical (Hiller and Goebel, 2007), which is a constrain of our animal model as we are not able to tell, if animals with a loud percept do also suffer more. Nevertheless, we do see tinnitus intensity and HL correlations in other studies with this animal model [e.g., (Lanaia et al., 2021)], which shows the principle approach to be valid. In this context, we recently proposed a new mechanism of tinnitus development based on neuronal stochastic resonance (SR) (Krauss et al., 2016a). It was proposed that, following noise trauma-induced HL, the SR adds neuronal noise to weak auditory signals at the level of the dorsal cochlear nucleus, thereby lifting otherwise sub-threshold signals above the response threshold of dorsal cochlear nucleus neurons so that the auditory information can be further propagated upstream the auditory pathway. In other words, the added noise would be “tuned up” in a frequency-specific manner to reduce noise trauma-induced HL. Simultaneously, the added noise signal is also propagated upstream the auditory pathway to the auditory cortex where it is finally perceived as tinnitus. In other words, tinnitus is the cost of the rescued hearing threshold (Krauss et al., 2018, 2019). This model of tinnitus development led to a number of predictions that, in the meantime, were demonstrated to be true: temporary auditory information depletion leads to partially reversible tinnitus (Krauss and Tziridis, 2021), chronic auditory information depletion leads to permanent tinnitus percepts (Tziridis et al., 2021), and patients with tinnitus have less HL compared to those without tinnitus (Gollnast et al., 2017). Interestingly, a linear correlation between HL and tinnitus strength has been described, indicating a direct link between HL and tinnitus development (Lanaia et al., 2021). However, to date, it is still unclear whether circadian effects (e.g., activity or hormonal levels) also play a role in either the tinnitus development itself or whether the tinnitus strength that develops after a HL is induced. Therefore, we aimed to investigate with this study if a noise trauma applied at different times of the day leads to hearing loss of different strengths in a diurnal species and if a thereby induced tinnitus development differs depending on the trauma induction time.

## Materials and Methods

### Ethical Statement

Mongolian gerbils (*Meriones unguiculatus*) were housed in type IV cages in a UniProtect Air Flow Cabinet (Zoonlab, Castrop-Rauxel, Germany, ambient noise level 55 dB SPL) in groups of 3 to 4 animals with free access to water and food (complete food for Gerbils, V1644-000, ssniff Spezialdiäten GmbH, Soest, Germany) at 24°C room temperature and 50% relative air humidity under 12-/12-h dark/light cycle (6 AM to 6 PM light). Animals had access to nesting material (paper nestlings) and other enrichments (cardboard tubes). The use and care of animals were approved by the state of Bavaria (Regierungspräsidium Unterfranken, Würzburg, Germany, no. 54.2.2-2532-2-540). A total of thirty-four 10–12-week-old (65 to 77 g) male gerbils purchased from Janvier (Saint Berthevin, France) were used in this study.

### Time Regime of Behavioral Testing, Auditory Brainstem Responses, and Acoustic Trauma

**Figure 1:**
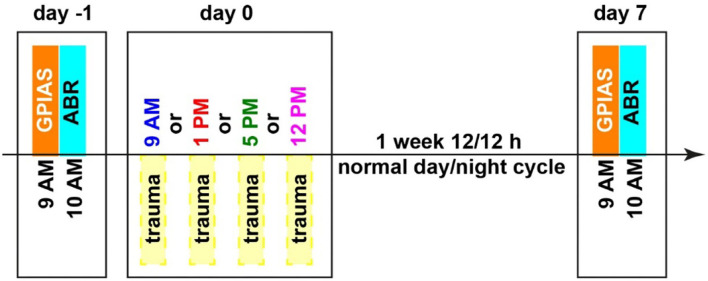
Time regime of behavioral testing for tinnitus assessment (GPIAS), hearing threshold measurements (ABR), and mild acoustic trauma (2 kHz, 115 dB SPL, 75 min). Different trauma times are depicted in different colors, which will also be used in the following figures. Zeitgeber times (ZT, time of light on) correspond as follows: 9 AM = ZT 3, 10 AM = ZT 4, 1 PM = ZT 7, 5 PM = ZT 11 and 12 PM = ZT 18.

All animals were handled before the beginning of the experiments and accustomed to the setup environment to minimize stress. Animals were separated into four groups with 8 to 10 animals each, animals were randomly allocated to the groups by the experimenter, and therefore, he was not blinded. The only difference between the groups was the time of the acoustic trauma (2 kHz, 115 dB SPL, 75 min) which either started at 9 AM, 1 PM, 5 PM, or 12 PM, no control group was included in this study. Relative to the Zeitgeber time (ZT), i.e., the onset of light was at 6 AM, this is ZT3, ZT7, ZT11, and ZT18. These daylight times were selected to span the light part of the activity cycle roughly equal, and the 12 PM time point was chosen as it was the center time of the dark cycle. All other measurements were taken at the identical time of the day. This means that the gap prepulse inhibition of the ASR gap-prepulse inhibition of the acoustic startle reflex (GPIAS) measurement for behavioral tinnitus assessment always started at 9 AM with a duration of 60 min, and the auditory brainstem response (ABR) measurements started at 10 AM with a duration of 30 min per ear and a randomized order of measurements for both ears. An overview of the timing is depicted in Figure 1, all behavioral and electrophysiological methods used have previously been described in detail (Ahlf et al., 2012; Tziridis et al., 2014, 2015; Krauss et al., 2016b; Schilling et al., 2017, 2019) and are therefore only described briefly here.

### Behavioral Testing (GPIAS)

The ASR is a widely used behavioral approach that does not require any training prior to testing. The original prepulse inhibition of the ASR has been modified into GPIAS to assess a possible tinnitus percept (Turner et al., 2006; Turner, 2007). In this study, animals were tested first with a gap–no-gap paradigm to measure the prepulse inhibition (PPI) of the startle response amplitudes usually 1 to 2 days before the acoustic trauma. These data were used as the healthy baseline condition for the calculation of the effect size of the change of PPI (cf. Statistical Analyses). For ASR measurement, animals were placed in a 15-cm-long, 4.2-cm-wide acrylic tube on a 3D-acceleration sensor platform in front of two speakers (one for the background stimulus and one for the startle pulse) inside a dark acoustic chamber (Schilling et al., 2017; Gerum et al., 2019). For adaptation, the animals were given 15-min rest, following which the stimulation started with five adaptation stimuli that were discarded from further analysis. The proper GPIAS stimuli consisted of 60 stimuli of band-pass filtered (digital 4^th^-order Butterworth filter, 48 dB/octave) background noise (10 s, 60 dB SPL) with center frequencies of 1, 2, 4, and 8 kHz and half an octave width each. They were presented with (30 stimuli) or without (30 stimuli) a 50-ms gap (2-ms sin^2^-ramps) 100 ms before a startle stimulus (white noise burst, 20-ms duration, 2-ms sin^2^-ramps, 105 dB SPL) in a pseudorandomized manner. A complete behavioral measurement of 240 stimuli had a duration of roughly 45 min. Additionally, 5 habituation stimuli at the beginning of the whole procedure were presented but not recorded. The exact same measurements were repeated 7 days after the acoustic trauma at the same time of the day (cf. Figure 1).

### Hearing Thresholds (ABR)

After the GPIAS measurement, the animals were anesthetized with 0.3 to 0.4 ml of a mixture of ketamine and xylazine (ketamine 500 mg/kg, xylazine 25 mg/kg, s.c.) and placed on a remote-controlled heating pad to obtain individual baseline audiograms (i.e., ABR) of each healthy animal (Supplementary Figure S1). The stimulation frequencies between 1 and 8 kHz in octave steps for stimulation intensities ranging from 0 to 90 dB SPL in 5 dB steps were presented free-field *via* a loudspeaker placed 3 cm in front of the pinna of the measured ear. For each ear, stimulus and intensity, 300 repetitions (each) of 6-ms-long phase-inverted double stimuli (100-ms intrastimulus interval, 500-ms interstimulus interval) were presented. The complete measurement of one ear took less than 30 min. For the ABR measurements, three silver electrodes were placed subcutaneously, one retro-aural above the bulla of the tested ear (recording electrode), another one central between both ears (reference electrode), and a last one at the basis of the tail (ground electrode). The signal was recorded and filtered (bandpass filter 400 to 2,000 Hz) *via* a Neuroamp 401 amplifier (JHM, Mainaschaff, Germany). As for the GPIAS, the exact same measurements were repeated 7 days after the acoustic trauma at the same time of the day.

### Acoustic Trauma

Then, 1 to 2 days after the baseline measurements of GPIAS and ABR, the animals were again anesthetized with the same ketamine–xylazine mixture and placed on a remote-controlled heating pad, 10 cm in front of a speaker inside a dark acoustic chamber. A mild binaural acoustic trauma was induced using a 2 kHz pure tone at 115 dB SPL lasting for 75 min. These adjustments were selected as they provided the highest rates of tinnitus development in our laboratory, with the trauma frequency being slightly below the best hearing range of the animals (Tziridis et al., 2014). The start of the sound exposure was either at 9 AM, 1 PM, 5 PM, or 12 PM to sample the two peaked circadian cycles of a male gerbil (Roper, 1976). In the 12 PM condition, animals were handled only under red light to prevent resetting of the circadian system, as mice and gerbils share the lack of red receptor cones (Rocha et al., 2016). The animals were usually sleeping for 120 min, and after fully waking up, they were returned to their home cage into the normal 12-/12-h light (6 AM to 6 PM)/dark (6 PM to 6 AM) rhythm.

### Statistical Analyses

The obtained data of the GPIAS and ABR measurements were evaluated objectively (i.e., automatically) by custom-made Python programs (Ahlf et al., 2012; Gollnast et al., 2017). The threshold determination was performed by an automated approach using the root mean square (RMS) values of the ABR amplitudes fitted with a hard-sigmoid function utilizing the background activity as offset (Schilling et al., 2019). The mean threshold was set at the level of slope change of this hard-sigmoid fit independent for each frequency and for each time point. HL was calculated by subtracting the post-trauma from the pre-trauma thresholds and tested by parametric tests. Positive values indicate worse hearing after the trauma, and negative values indicate better hearing. The HL was calculated separately for each ear, and the ear with less HL was called the less affected ear and the other the stronger affected ear. Mean HL is the mean binaural HL for each animal. For statistical evaluation, two-factorial ANOVAs with Tukey's *post hoc* tests were performed. Additionally, we investigated as a kind of *post hoc* test one-factorial ANOVAs at different stimulation frequencies to “zoom in” on possible temporal threshold effects of the trauma. Significant hearing loss was also tested by Bonferroni-corrected single sample *t*-tests vs. zero.

Tinnitus development was tested for each animal and frequency individually by *t*-tests of the log-normalized PPI: The log-normalization of the response amplitudes of gap (A_gap_) and no-gap (A_no−gap_) by log(A_gap_/A_no−gap_) is necessary as it has been shown that, only after this calculation, parametrical testing (e.g., by *t*-tests) is allowed (Schilling et al., 2017). A significant decrease (*p* < 0.05, Bonferroni-corrected) of the GPIAS-induced change of the response to the ASR after the trauma (GPIAS_post_) relative to conditions before the trauma (GPIAS_pre_) in that test was rated as an indication of a tinnitus percept at that specific frequency. The effect size of the log-normalized PPI change can be interpreted as a correlate of the subjective tinnitus loudness. With this behavioral approach, we can group animals independent of any other measurements into those with behavioral signs of a tinnitus percept and those without (Schilling et al., 2017). The resulting distributions were tested by a chi-squared test for multiple groups.

Gap-prepulse inhibition of the acoustic startle reflex effect size was assessed by the Kruskal–Wallis ANOVAs independent for each time of trauma induction. ABR threshold changes (less affected ear, stronger affected ear, and mean HL) were assessed independently from the GPIAS analyses by two-factorial ANOVAs (factors *stimulation frequency* and *time of trauma*). Correlations of behavior and HL were calculated with multiple linear regression analyses for the four different time points separately. Additionally, the behavioral data were correlated with the mean HL and the individual HL of the two differently affected ears.

## Results

### Hearing Loss Induced by Noise Trauma at Different Times of the Day

**Figure 2:**
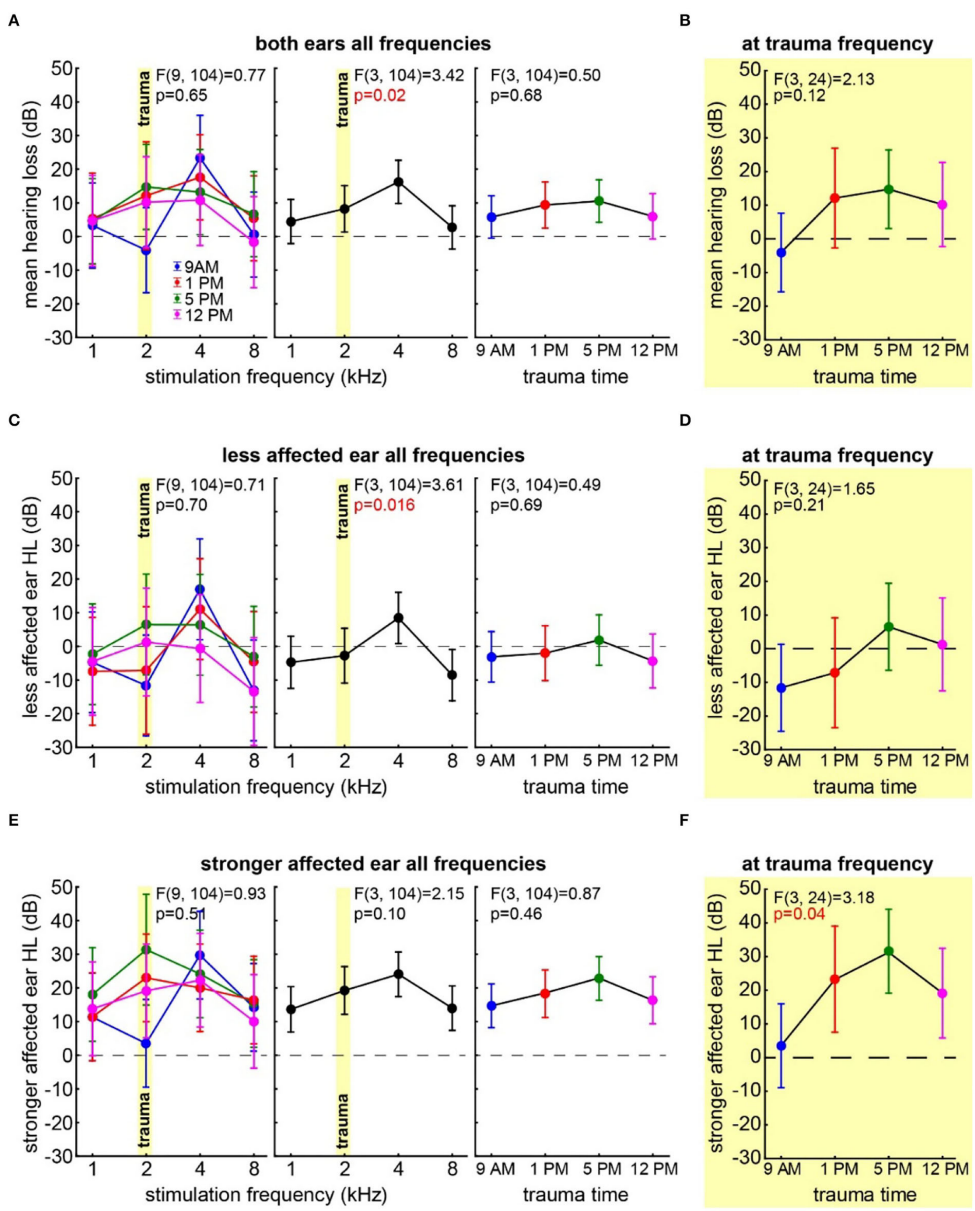
Hearing loss one week after trauma at different times of the day. **(A)** Mean HL across both ears analyzed by a two-factorial ANOVA with factors *time of day* (right panel) and *frequency* (center panel), the interaction of both factors is depicted in the left panel, *time of day* is color-coded, the trauma frequency is marked by a yellow bar, and whiskers give the 95% confidence intervals, significant *p*-values of the F-statistics are red. **(B)** One-factorial ANOVA of the mean HL of both ears at the trauma frequency over the *time of day*. **(C)** Two-factorial ANOVA of the HL of the less affected ear with factors *time of day* and *frequency*. **(D)** One-factorial ANOVA of the HL of the less affected ear at the trauma frequency over the *time of day*. **(E)** Two-factorial ANOVA of the HL of the stronger affected ear with factors *time of day* and *frequency*. **(F)** One-factorial ANOVA of the HL of the stronger affected ear at the trauma frequency over the *time of day*.

First, we investigated the mean binaural HL induced by the trauma at the four different times of the day by a two-factorial ANOVA with the factors *time of day* and *frequency*. Figure 2A depicts the results of this analysis. It shows no significant *time* effect averaged across all frequencies (also cf. Table 1) on the mean HL of both ears (right panel: F_(3, 104)_ = 0.50, *p* = 0.68) but a significant *frequency* effect averaged across all times (center panel: F_(3, 104)_ = 3.42, *p* = 0.02), indicating that the pure tone trauma induced a frequency-specific HL. The *interaction* of both factors revealed no significant differences in the mean binaural HL over the four times of the day and the four frequencies (F_(9, 104)_ = 0.77, *p* = 0.65). In other words, binaural HL was comparable in all four groups, resulting in a frequency-specific HL around 4 kHz with the biggest variances seemingly around the trauma and the 4 kHz frequencies. This is also the case when grouping the data in only two-time groups, “early” (9 AM and 1 PM) and “late” (5 PM and 12 PM), for a higher statistical power as can be seen in Supplementary Figure S2A. In Figure 2B, the one-factorial ANOVA of the mean binaural HL at the trauma frequency over the different *times of day* of the trauma is given. No significant effect could be found (F_(3, 24)_ = 2.13, *p* = 0.12), indicating that the mean binaural HL at the trauma frequency was comparable between the four trauma groups. Nevertheless, we found a significant HL (single sample *t*-test vs. 0) in the 5 PM trauma group only (mean ± standard deviation: 14.7 ± 15.3 dB, *p* = 0.03). For the most affected frequency of 4 kHz, we also did not see any time dependency of the mean binaural HL on the *time* of trauma by a 1-factorial ANOVA (F_(3, 24)_ = 0.41, *p* = 0.75), and the single sample *t*-test vs. Also, 0 only revealed a significant HL at 5 PM (17.61 ± 9.71 dB, *p* = 0.001). In conclusion, there might be a trend for higher HL values in binaural HL analysis when the trauma was applied at 5 PM, but the effect is not very prominent.

**Table 1:** Statistics of one-factorial ANOVAs of HL over the different *frequencies* and single sample *t*-tests vs. 0 in the four trauma time conditions.

**Trauma condition**	**Frequency-dependent F statistics**	**Frequency-dependent *p*-value**	**General effect: mean ±standard deviation**	**General effect: single-sample *t*-test vs. 0 *p*-value**
9 AM	F_(3, 28)_ = 3.21	0.038	14.70 ± 21.00	0.14
1 PM	F_(3, 28)_ = 0.40	0.228	17.71 ± 21.11	0.07
5 PM	F_(3, 24)_ = 1,55	0.754	21.41 ± 14.22	0.01
12 PM	F_(3, 24)_ = 1.00	0.409	16.33 ± 17.15	0.06

Next, we focused our analyses on the two individual ears of each animal separately. In all animals, one ear showed a lower mean HL (less affected ear) compared to the other (stronger affected ear). In the less affected ear (Figure 2C), we found a similar result for the two-factorial ANOVA of the mean HL as in the binaural analysis, with no significant effect of the *time of day* the trauma was applied (F_(3, 104)_ = 0.49, *p* = 0.69), a significant *frequency* effect (F_(3, 104)_ = 3.61, *p* = 0.016), and no significant *interaction* of both factors (F_(9, 104)_ = 0.71, *p* = 0.70). Also, in the one-factorial ANOVA of the mean HL of the less affected ear at the trauma frequency (Figure 2D), no significant differences between the HL induced by the trauma at different *times of the day* could be found (F_(3, 24)_ = 1.65, *p* = 0.21). Note that the less affected ear shows hardly any change relative to the pre-trauma condition, and no mean HL value at any point in time was significantly different from zero. In the strongest affected frequency of 4 kHz (cf. Figure 2C, center panel), we did not see any *time* dependency in the HL of the less affected ear (one-factorial ANOVA: F_(3, 24)_ = 0.61, *p* = 0.62), and the single sample *t*-tests vs. 0 only showed a significant HL at 5 PM (11.07 ± 10.57 dB, *p* = 0.03). Taken together, the less affected ear did show a frequency-dependent HL around 4 kHz, but also one could only speak of a trend that the HL in the 5 PM trauma group might be stronger compared to the other groups.

The stronger affected ear on the other hand showed a somewhat different pattern (Figure 2E), as neither factor *time of day* (F_(3, 104)_ = 0.87, *p* = 0.46) nor *frequency* (F_(3, 104)_ = 2.15*, p* = 0.10) nor *interaction* (F_(9, 104)_ = 0.93, *p* = 0.51) showed any kind of significant effect on the mean HL in that ear. Note that in the factor *frequency*, a weak tendency could be found. Nevertheless, contrary to the other two analyses, we did not see strong differences in the HL in the factor *frequency* in these ears (cf. Figures 2A,E, center panels). For comparison with the less affected ear and the mean binaural HL (i.e., the two analyses above), we compared the effect of the *time* of the trauma also at the trauma frequency of 2 and at 4 kHz by two one-factorial ANOVAs. In the first one-factorial ANOVA of the mean HL at the trauma frequency of the stronger affected ear (Figure 2F), we found a significant effect of the *time of the day* of the trauma induction with F_(3, 24)_ = 3.18, *p* = 0.04 and significant mean HL values (single sample *t*-test vs. 0) at 1 PM (*p* = 0.013), 5 PM (*p* = 0.005), and 12 PM (*p* = 0.036). In the second one-factorial ANOVA at 4 kHz, we found no time dependency of the HL of the stronger affected ear (F_(3, 24)_ = 0.24, *p* = 0.87), but except for the 1 PM trauma, we found time significant hearing losses with single sample *t*-tests vs. 0 (9 AM: 29.73 ± 28.23 dB, *p* = 0.02, 5 PM: 24.15 ± 10.84 dB, *p* < 0.001, 12 PM: 22.29 ± 19.48 dB, *p* = 0.02). In other words, the HL in the stronger affected ear was greater in the quality of a general threshold increase, but in a specific frequency, the ranges significantly depended on the trauma time.

### Effect Size of Behavior Change by Trauma at Different Times of the Day

**Figure 3:**
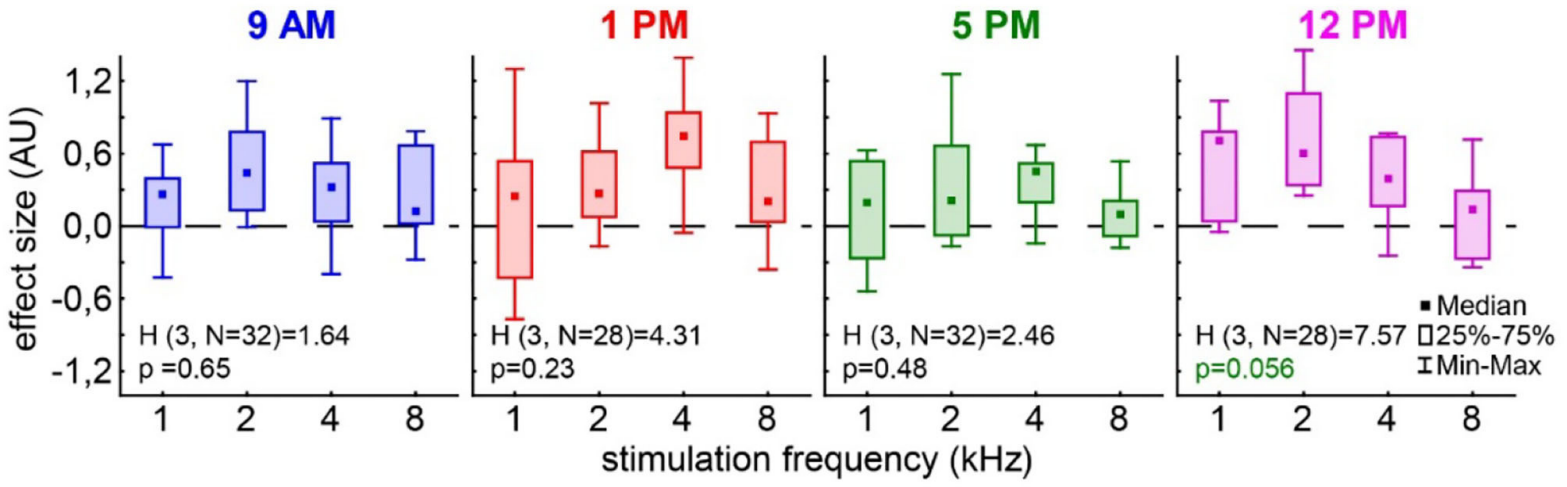
Effect size of the PPI change post vs. pre-trauma. The Kruskal–Wallis ANOVAs (H-statistics) were calculated independently for each trauma time group. Boxes depict the interquartile range, and whiskers give the range of the complete distribution.

**Figure 4:**
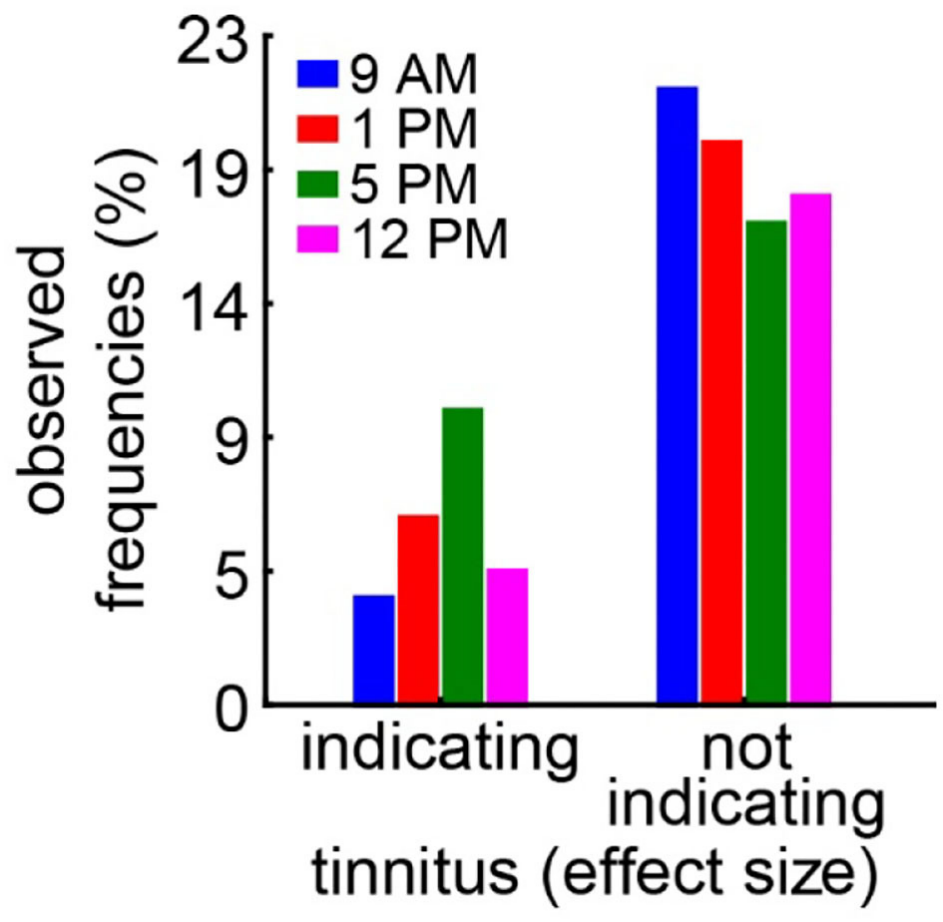
Relative number of frequencies with or without behavioral signs of a tinnitus percept. A significantly negative effect size indicates a possible tinnitus percept and is counted as such. A chi-squared test for multiple groups (*p* = 0.23) reveals no significant difference in the response distributions.

In the case of the tinnitus assessment, we could not disentangle the effects of the single ears, as we measured the behavior of the awake animal. First, we analyzed the median effect size of the response change at the different frequencies of the four different trauma time groups independently by the Kruskal–Wallis ANOVAs (Figure 3). The median effect size represents the overall response of all animals and gives a general overview of the change of the PPI responses post- vs. pre-trauma but not the individual percept of each animal. Note that only negative values indicate a possible tinnitus percept at a given frequency. In this general overview, we found no significant differences in the responses to the different frequencies at 9 AM (H_(3, 32)_ = 1.64, *p* = 0.65), 1 PM (H_(3, 28)_ = 4.31, *p* = 0.23), or 5 PM (H_(3, 32)_ = 2.46, *p* = 0.48), but at least a strong tendency for such a frequency-specific effect on the effect size of the PPI difference at 12 PM with H_(3, 28)_ = 7.57 and *p* = 0.056. A comparable result was found, when the data were grouped into only two trauma time groups, i.e., early and late (Supplementary Figure S2B). No significant difference emerged in the early group (H _(3, N = 60)_ = 4.31, *p* = 0.23) whereas the late statistics showed a tendency for a frequency dependency of the effect size (H _(3, N = 60)_ = 6.85, *p* = 0.077). To estimate the frequency of individual appearance of tinnitus percepts across animals in the four tested frequency ranges, we counted the number of significant negative effect sizes at each frequency and trauma time condition and plotted them as a percentage of complete responses in Figure 4. In only 26% of all responses, a tinnitus percept was found with a peak of 10% of all responses after trauma at 5 PM. Note that a noise trauma at 5 PM also indicated the strongest HL. Nevertheless, a chi-squared test for multiple groups revealed no significant differences (X^2^_(3, 104)_ = 4.31, *p* = 0.23) between the group response distributions. In other words, when only looking at the behavioral changes related to possible tinnitus percepts, we could not identify any significant dependencies on the time of trauma.

### Linear Regression Analyses of Hearing Loss and Effect Size

**Figure 5:**
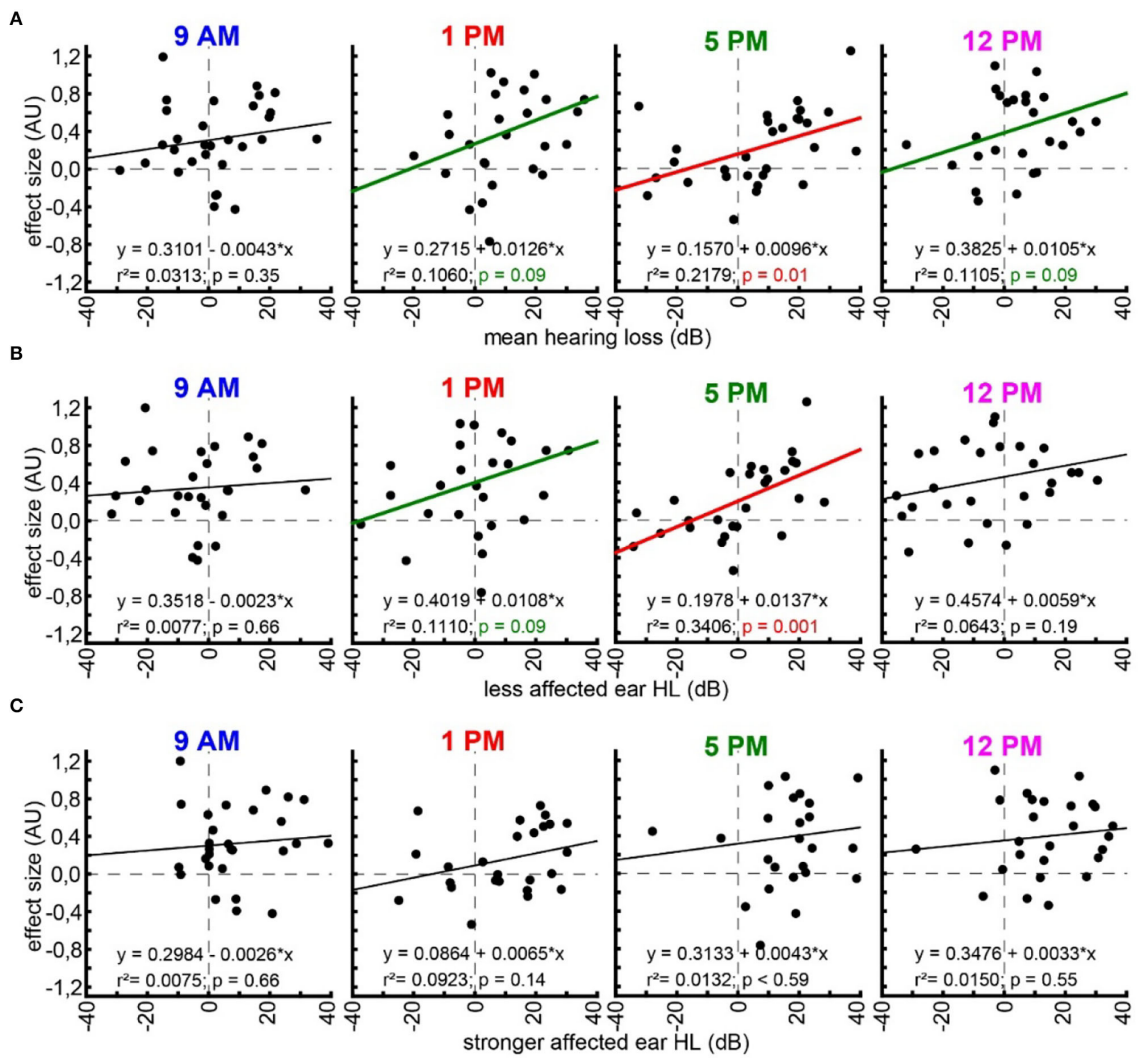
Linear regression analyses of hearing loss and effect size. **(A)** Multiple linear regressions for effect size and mean binaural hearing loss for each trauma time group (panels from left to right). Linear regression formula, r^2^-value, and *p*-value are given for each case. Black regression lines and *p*-values indicate no significant regression, green indicated a tendency, and red indicates a significant regression. **(B)** Multiple linear regressions for effect size and less affected ear hearing loss. **(C)** Multiple linear regressions for effect size and stronger affected ear hearing loss.

With the idea of the model of SR for tinnitus development (cf. Introduction) in mind, we finally investigated the relationship between hearing loss (which is believed to trigger SR) and the tinnitus percept (a consequence of SR) at the given frequencies by multiple linear regression analyses (Figure 5). First, we looked at the relationship between the frequency-matched values of the mean binaural hearing loss and the behavioral effect size in the four different trauma time groups (Figure 5A). We found a significant linear regression in the 5 PM trauma group with r^2^ = 0.22 and *p* = 0.01. In other words, higher HL values led to more values with positive effect sizes in that group, while hearing threshold gain correlates with negative effect size values, indicating a tinnitus percept. Additionally, in the 1 PM as well as in the 12 PM trauma group, we see tendencies for such a linear correlation, both with r^2^-values around 0.11 and *p* = 0.09. When grouping the data into only two trauma time groups (early and late), the results were even more clear with the early trauma animals showing no significant correlation between HL and effect size with r^2^ = 0.02 and *p* = 0.26 and the late animals showing such a correlation with r^2^ = 0.12 and *p* = 0.007 (Supplementary Figure S2C). In the less affected ears (Figure 5B), we found an even stronger correlation between HL and effect size (r^2^-value of 0.34 and a *p* = 0.001) in the 5 PM trauma group but only a tendency for a linear correlation of both variables in the 1 PM trauma group (r^2^ = 0.11, *p* = 0.09). Finally, in the stronger affected ears (Figure 5C), we did not observe any significant correlation between the two variables. Note that most of the HL values in these ears were positive, indicating a real hearing loss.

**Figure 6:**
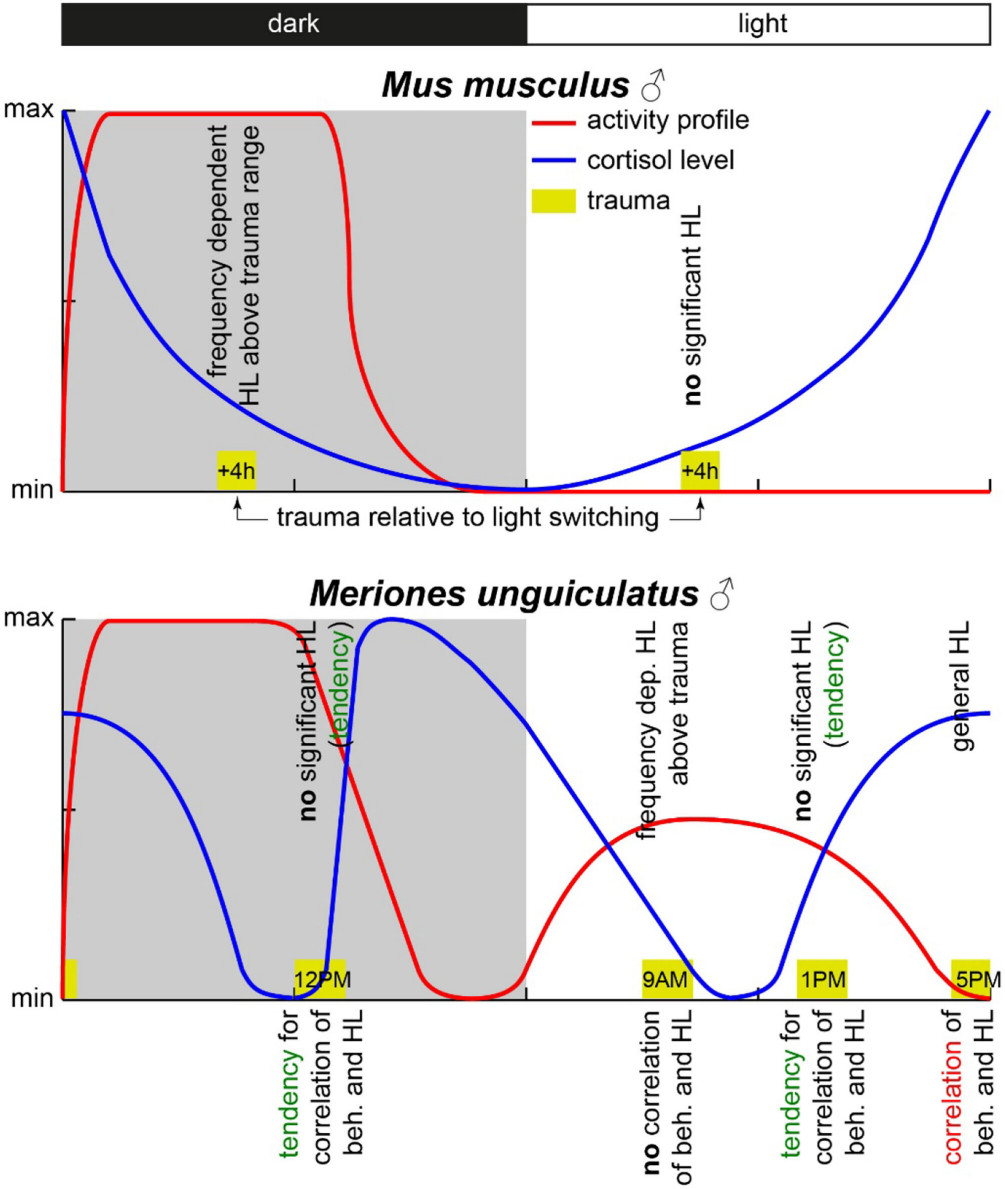
Scheme of activity profiles and blood cortisol concentration in mice and Mongolian gerbils. The yellow boxes mark the times of trauma with the start times given inside. The observed HL effects are given above the trauma boxes, and the observed correlation between behavioral changes and HL is given below the respective trauma symbol. Data for this scheme are derived from (Roper, 1976; Saito et al., 1989; Valentinuzzi et al., 1997; Meltser et al., 2014; Hu et al., 2020) and this study.

Concluding the results, we were able to prove that only the combinatory view of hearing loss and the behavioral correlates of a tinnitus percept showed the circadian effects of acoustic trauma in detail, whereas each variable alone did show only minor temporal dependencies. However, contrary to our hypothesis, the strongest and most general effects found were in the afternoon, when the activity of the animals was minimal (cf. Figure 6).

## Discussion

In this study, we aimed to investigate, first, whether noise trauma applied at different times of the day leads to hearing loss of different strengths in a diurnal species, the Mongolian gerbil, and, second, if tinnitus development induced by such noise trauma differs depending on trauma induction time. We could show only minor effects in the single variables, but in the combination, we found clear correlations between HL and behavioral correlates of tinnitus at different time points. When analyzing the variables independently, we found that, especially around the trauma frequency, the stronger affected ear showed a significant dependency of the HL on the time of the trauma with the amount of HL peaking around 5 PM – corresponding to the end of the activity cycle in these animals. The rate of observed tinnitus frequencies also tended to be highest around this time of the day (i.e., 5 PM) even though we were not able to see any significant differences in the effect sizes. When correlating the individual tinnitus-related behavioral changes with the individual changes in the hearing thresholds, we could show a clear correlation between tinnitus strength and hearing loss. This correlation was not seen for all trauma induction times but was peaked at 5 PM, i.e., the time of minimal activity of the animals (cf. below). Surprisingly, this correlation seems to be present not only in the less affected ears but also in mean binaural hearing loss values.

Before going into a detailed discussion, we would like to state that the study has several limitations. First of all, we did not use sham trauma groups for control, as this would need a sham group for each time point. Therefore, we only compared the effects of the trauma at different time points. Second, no histology was performed to investigate the effects of the trauma on the cochleae, as this was not the focus of this study. Nevertheless, we could already observe that tinnitus is most probably based on a synaptopathy of the ribbon synapses of the inner hair cells of the cochlea (Tziridis et al., 2021) and have no doubt that we would find similar results also in these animals. Third, we always used mild traumata to induce a maximum amount of tinnitus frequencies in our studies [e.g., (Ahlf et al., 2012; Tziridis et al., 2014, 2015; Krauss et al., 2016b)]. As it has been speculated for other rodents, that mild (or too strong) traumata might be not optimal for circadian studies, as the effects might be too small to be detected or might be dampened by “noise” (Fontana et al., 2019; Sheppard et al., 2019). We agree with this view on the level of single variables, but as we could see in this study, by combining the relevant variables, a strong effect can be shown even in mild acoustic trauma data. Finally, the circadian behavior of gerbils is clearly different from other rodents (cf. below and Figure 6). For example, the foraging behavior of animals is usually spread over the activity period, i.e., for mice, this is the night butthis is the whole day for gerbils. Eating has a clear impact on clock rhythms in several organs (Gachon et al., 2004; Mohawk et al., 2012) and might therefore be one of the uncontrolled factors that affect the outcome of our but also other studies.

In nocturnal mice, the most prominent effect on HT after noise trauma was found when broadband noise trauma was applied during their active phase (Meltser et al., 2014), but during their resting period during the day, the animals showed hardly any impairment in hearing after 2 weeks of recovery [for other acoustic trauma-related circadian effects and overview cf. also (Kiefer et al., 2022)]. In this study with Mongolian gerbils, we found the most prominent effects after 1 week after a 2 kHz pure tone trauma around the end of their activity cycle, i.e., near the end of the light phase. For an easier comparison of the two animal models, we present a synopsis of the activity profiles and corresponding blood cortisol levels at the respective trauma times in Figure 6. The circadian rhythm within the cochlea is, among others, controlled *via* the suprachiasmatic nucleus and glucocorticoids (Lightman and Conway-Campbell, 2010). The rough scheme presented here is based on data from five different studies in the literature (Roper, 1976; Saito et al., 1989; Valentinuzzi et al., 1997; Meltser et al., 2014; Hu et al., 2020). The peak activity of mice starts with the beginning of the dark cycle and lasts roughly 7 to 8 h (Siepka and Takahashi, 2005), whereas in male gerbils, an activity cycle with two peaks has been described (Roper, 1976). One activity phase started roughly 1 h before the beginning of the light phase (in our case around 5 AM), peaking around 5 h into the light phase (here: 11 AM) and having a trough at the beginning of the dark cycle (here: 6 PM). After that, the activity peaked again 4 h into darkness (here: 10 PM) and then went back to the second trough 1 h before the light cycle starts again. In our approach, we applied the trauma either 2 h before (9 AM) or 2 h after (1 PM) the daytime peak activity as well as near the activity trough at the end of the light cycle (5 PM) and mid-night activity at 12 PM. In contrast to mice, the strongest trauma effects were found around the activity trough at 5 PM (Table 1) with the stronger affected ear showing a very broad HL and explicitly loosing at 2 kHz around 27 dB. Also, the averaged hearing impairment of both ears still reached 14 dB at 5 PM, whereas the values of the 9 AM trauma showed an extremely focused hearing impairment, only affecting the 4 kHz frequency range (mean binaural HL (9 AM) one-factorial ANOVA: F_(3, 28)_ = 3.21, *p* = 0.038, Tukey's *post hoc* 2 kHz vs. 4 KHz: *p* = 0.037). In other words, we saw different HL patterns when applying an acoustic trauma at different times of the day. These patterns are more complex than those in mice, where trauma during the peak activity leads to strong hearing impairment. This higher complexity in the gerbil compared to mice may be because the activity cycle in gerbils with its two activity peaks is more complex than the cycle in mice that shows only one activity peak. In general, the different trauma effects in gerbils could be due to several different reasons as the circadian rhythms affect the different organ systems differently. One probable candidate for mediating the described effect is cortisol (Figure 6). In mice, the peak cortisol levels in the blood are found at the beginning of the dark cycle (Gong et al., 2015) corresponding to the strong effect the trauma has on the animals during that time. In gerbils, the cortisol levels were at a minimum at the beginning of the light cycle and then were roughly stable over the day (Liu et al., 2019) well into the dark phase. When looking at the described activity patterns and the blood cortisol levels of mice and gerbils at the different trauma times, a pattern emerges. We found a frequency-specific effect of the trauma on the hearing thresholds of the two animal species at times when activity is high and cortisol levels are dropping. We found no significant general HL (even though we see tendencies for this in Mongolian gerbils) when the activity is low or at least dropping (this could be a possible reason for described tendencies in gerbils) and blood cortisol levels are rising. Finally, in gerbils, we found a general, not frequency-specific, HL when activity is low and blood cortisol levels are high (not tested in mice).

The second question we asked is whether the different trauma times also lead to the differences in tinnitus development following the trauma. It is well accepted that, in humans, tinnitus can be a consequence of hearing loss [e.g., (Heller, 2003; Pan et al., 2009; Boussaty et al., 2021; Tziridis et al., 2021)] and that tinnitus severity and hearing loss intensity in patients with noise-induced hearing loss correlate (Searchfield et al., 2007; Mazurek et al., 2010), but it is unknown whether different trauma times also play a role in its development. Our approach here was the analysis of the effect size of the PPI change post- vs. pre-trauma (Schilling et al., 2017). It enabled us not only to detect a possible tinnitus percept at a given frequency range (± ½ octave around a center frequency) but also calculate a relative tinnitus strength, with the more negative values representing a louder percept (Krauss and Tziridis, 2021; Lanaia et al., 2021). Positive values are most likely the result of cortical processing and learning (Moreno-Paublete et al., 2017) that counteract any negative value of the effect size. In other words, we most probably underestimate the average number of tinnitus percepts, which may also explain the strong positive offsets of the calculated linear regressions shown in Figure 5 and Supplementary Figure S2C. Independent of this possible offset, the method has the advantage of being very fast as it does not require any learning but is hard to cross-validate and is therefore debatable (Eggermont, 2013). Nevertheless, in the effect size data (Figure 3), we found no strong differences in the PPI response changes to the different frequencies relative to the different times of the pure tone trauma applied. The tendency found for such a difference was in the effect size data for the trauma at 12 PM. When looking into the details of the analysis, it became clear that this possible difference was a more positive value at 2 kHz, indicating even less tinnitus/more cortical learning under that specific condition. In addition, the counts of the significant tinnitus frequencies (Figure 4) did not show any significant differences between the tinnitus/non-tinnitus frequency ratios. Therefore, in the tinnitus strength or numbers, we could not find any trauma time dependency.

This changed when correlating both variables with each other based on our hypothesis of SR-dependent tinnitus development. This hypothesis assumes that the information flow from the auditory periphery to the central nervous system is constantly optimized by the well-described neurobiological mechanism of SR (Shannon, 1948; Gammaitoni et al., 1998) (cf. Introduction). The data presented here provide an additional aspect of the correlation of both variables (Figure 5) as it became clear that the correlation between tinnitus loudness and hearing loss is most prominent when the trauma is applied at the end of the light cycle (i.e., end of activity phase) at 5 PM and the behavior is correlated with the less affected ear. This could indicate that the perceived tinnitus may be monaural at least in the majority of the investigated animals and/or only smaller hearing losses can be rescued by the proposed mechanism as suggested by our study and others (Zeng et al., 2000; Schilling et al., 2020). The fact that the correlation between HL and tinnitus-related behavior was dependent on the trauma induction time indicates that HL is not the only factor that determines tinnitus development but that the tinnitus developing mechanism itself is influenced by the time of trauma induction. In other words, tinnitus development has a circadian component irrespective of noise trauma severity.

## Conclusions

We observed that the time of acoustic trauma plays a significant role not only in the amount of hearing loss it inflicts but also in the strength of a subsequent development of a tinnitus percept. Diurnal as well as nocturnal animals seem to show a similar pattern as the vulnerability for a frequency-dependent hearing loss seems to be highest around their peak activity phase, while at lower activities, there is either no significant impairment or a more generalized effect on all frequencies. The development of tinnitus follows this pattern, in line with the hypothesis that the impairment is the source of the development of the percept. For future investigations – also in humans – it might be crucial to take these circadian effects into account.

## Data Availability Statement

The raw data supporting the conclusions of this article will be made available by the authors, without undue reservation.

## Ethics Statement

The animal study was reviewed and approved by Regierungspräsidium Unterfranken, Würzburg, Germany, No. 54.2.2-2532-2-540.

## Author Contributions

KT and HS planned the experiments and wrote the manuscript. JG performed all experiments. JG and KT performed the statistical analyses. All authors contributed to the article and approved the submitted version.

## Conflict of Interest

The authors declare that the research was conducted in the absence of any commercial or financial relationships that could be construed as a potential conflict of interest.

## Publisher's Note

All claims expressed in this article are solely those of the authors and do not necessarily represent those of their affiliated organizations, or those of the publisher, the editors and the reviewers. Any product that may be evaluated in this article, or claim that may be made by its manufacturer, is not guaranteed or endorsed by the publisher.

## References

[B1] Ahlf S. Tziridis K. Korn S. Strohmeyer I. Schulze H. (2012). Predisposition for and prevention of subjective tinnitus development. PLoS ONE. 7, e44519. 10.1371/journal.pone.0044519 23056180 PMC3462765

[B2] Boussaty E. C. Friedman R. A. Clifford R. E. (2021). Hearing loss and tinnitus: association studies for complex-hearing disorders in mouse and man. Human Genet. 141, 1–10. 10.1007/s00439-021-02317-9 34318347 PMC8792513

[B3] Eggermont J. J. (2013). Hearing loss, hyperacusis, or tinnitus, what is modeled in animal research? Hear. Res. 295, 140–149. 10.1016/j.heares.2012.01.005 22330978

[B4] Fontana J. M. Tserga E. Sarlus H. Canlon B. Cederroth C. (2019). Impact of noise exposure on the circadian clock in the auditory system. J. Acoust. Soc. Am. 146, 3960–3966. 10.1121/1.5132290 31795664 PMC7341678

[B5] Frankland P. W. Ralph M. R. (1995). Circadian modulation in the rat acoustic startle circuit. Behav. Neurosci. 109, 43. 10.1037/0735-7044.109.1.43 7734079

[B6] Gachon F. Nagoshi E. Brown S. A. Ripperger J. Schibler U. (2004). The mammalian circadian timing system, from gene expression to physiology. Chromosoma. 113, 103–112. 10.1007/s00412-004-0296-2 15338234

[B7] Gammaitoni L. Hänggi P. Jung P. Marchesoni F. (1998). Stochastic resonance. Rev. Mod. Phys. 70, 223. 10.1103/RevModPhys.70.223

[B8] Gerum R. C. Rahlfs H. Streb M. Krauss P. Grimm J. Metzner C.. (2019). Open (G) PIAS, An open-source solution for the construction of a high-precision acoustic startle response setup for tinnitus screening and threshold estimation in rodents. Front. Behav. Neurosci. 13, 140. 10.3389/fnbeh.2019.00140 31293403 PMC6603242

[B9] Goel N. (2005). Late-night presentation of an auditory stimulus phase delays human circadian rhythms. Am. J. Physiol. Regul. Integr. Compar. Physiol. 289, R209–R216. 10.1152/ajpregu.00754.2004 15790749

[B10] Gollnast D. Tziridis K. Krauss P. Schilling A. Hoppe U. Schulze H. (2017). Analysis of audiometric differences of patients with and without tinnitus in a large clinical database. Front. Neurol. 8, 31. 10.3389/fneur.2017.00031 28232817 PMC5298966

[B11] Gong S. Miao Y. L. Jiao G. Z. Sun M. J. Li H. Lin J.. (2015). Dynamics and correlation of serum cortisol and corticosterone under different physiological or stressful conditions in mice. PLoS ONE. 10, e0117503. 10.1371/journal.pone.0117503 25699675 PMC4336318

[B12] Harrison R. T. (2019). Effect of Changes to the Circadian Rhythm on Susceptibility to Noise-and Drug-Induced Hearing Losses. Columbus, OH: The Ohio State University.

[B13] Heller A. J. (2003). Classification and epidemiology of tinnitus. Otolaryngol. Clin. North Am. 36, 239–248. 10.1016/S0030-6665(02)00160-3 12856294

[B14] Hiller W. Goebel G. (2007). When tinnitus loudness and annoyance are discrepant, audiological characteristics and psychological profile. Audiol. Neurotol. 12, 391–400. 10.1159/000106482 17664870

[B15] Hu H. Kang C. Hou X. Zhang Q. Meng Q. Jiang J.. (2020). Blue light deprivation produces depression-like responses in mongolian gerbils. Front. Psychiatry. 11, 233. 10.3389/fpsyt.2020.00233 32322220 PMC7156555

[B16] Kiefer L. Koch L. Merdan-Desik M. Gaese B. H. Nowotny M. (2022). Comparing the electrophysiological effects of traumatic noise exposure between rodents. J. Neurophysiol. 10.1152/jn.00081.2021 35020518

[B17] Konopka R. J. Benzer S. (1971). Clock mutants of Drosophila melanogaster. Proc Nat Acad Sci. 68, 2112–2116. 10.1073/pnas.68.9.2112 5002428 PMC389363

[B18] Krauss P. Schilling A. Tziridis K. Schulze H. (2019). Models of tinnitus development: from cochlea to cortex. HNO 67, 172–177. 10.1007/s00106-019-0612-z 30707242

[B19] Krauss P. Tziridis K. (2021). Simulated transient hearing loss improves auditory sensitivity. Sci. Rep. 11, 14791. 10.1038/s41598-021-94429-5 34285327 PMC8292442

[B20] Krauss P. Tziridis K. Buerbank S. Schilling A. Schulze H. (2016b). Therapeutic value of ginkgo biloba extract EGb 761(R) in an animal model (Meriones unguiculatus) for noise trauma induced hearing loss and tinnitus. PLoS ONE. 11, e0157574. 10.1371/journal.pone.0157574 27315063 PMC4912078

[B21] Krauss P. Tziridis K. Metzner C. Schilling A. Hoppe U. Schulze H. (2016a). Stochastic resonance controlled upregulation of internal noise after hearing loss as a putative cause of tinnitus-related neuronal hyperactivity. Front. Neurosci. 10, 597. 10.3389/fnins.2016.00597 28082861 PMC5187388

[B22] Krauss P. Tziridis K. Schilling A. Schulze H. (2018). Cross-modal stochastic resonance as a universal principle to enhance sensory processing. Front. Neurosci. 12, 578. 10.3389/fnins.2018.00578 30186104 PMC6110899

[B23] Lanaia V. Tziridis K. Schulze H. (2021). Salicylate-induced changes in hearing thresholds in mongolian gerbils are correlated with tinnitus frequency but not with tinnitus strength. Front. Behav. Neurosci. 15, 698516. 10.3389/fnbeh.2021.698516 34393736 PMC8363116

[B24] Lightman S. L. Conway-Campbell B. L. (2010). The crucial role of pulsatile activity of the HPA axis for continuous dynamic equilibration. Nat. Rev. Neurosci. 11, 710–718. 10.1038/nrn2914 20842176

[B25] Liu X. Zheng X. Liu Y. Du X. Chen Z. (2019). Effects of adaptation to handling on the circadian rhythmicity of blood solutes in Mongolian gerbils. Animal Models Exper. Med. 2, 127–131. 10.1002/ame2.12068 31392306 PMC6600653

[B26] Mazurek B. Olze H. Haupt H. Szczepek A. J. (2010). The more the worse, the grade of noise-induced hearing loss associates with the severity of tinnitus. Int. J. Environ. Res. Public Health. 7, 3071–3079. 10.3390/ijerph7083071 20948948 PMC2954569

[B27] Meltser I. Cederroth C. R. Basinou V. Savelyev S. Lundkvist G. S. Canlon B. (2014). TrkB-mediated protection against circadian sensitivity to noise trauma in the murine cochlea. Curr. Biol. 24, 658–663. 10.1016/j.cub.2014.01.047 24583017 PMC3962718

[B28] Miller M. W. Gronfier C. (2006). Diurnal variation of the startle reflex in relation to HPA-axis activity in humans. Psychophysiology. 43, 297–301. 10.1111/j.1469-8986.2006.00400.x 16805869 PMC3035930

[B29] Mohawk J. A. Green C. B. Takahashi J. S. (2012). Central and peripheral circadian clocks in mammals. Annu. Rev. Neurosci. 35, 445–462. 10.1146/annurev-neuro-060909-153128 22483041 PMC3710582

[B30] Moore R. Y. Eichler V. B. (1972). Loss of a circadian adrenal corticosterone rhythm following suprachiasmatic lesions in the rat. Brain Res. 42. 10.1016/0006-8993(72)90054-6 5047187

[B31] Moreno-Paublete R. Canlon B. Cederroth C. R. (2017). Differential neural responses underlying the inhibition of the startle response by pre-pulses or gaps in mice. Front. Cell Neurosci. 11, 19. 10.3389/fncel.2017.00019 28239338 PMC5302757

[B32] Pan T. Tyler R. S. Ji H. Coelho C. Gehringer A. K. Gogel S. A. (2009). The relationship between tinnitus pitch and the audiogram. Int. J. Audiol. 48, 277–294. 10.1080/14992020802581974 19842803

[B33] Park J. S. Cederroth C. R. Basinou V. Meltser I. Lundkvist G. Canlon B. (2016). Canlon, Identification of a circadian clock in the inferior colliculus and its dysregulation by noise exposure. J. Neurosci. 36, 5509–5519. 10.1523/JNEUROSCI.3616-15.2016 27194331 PMC4871986

[B34] Richards J. Gumz M. L. (2013). Mechanism of the circadian clock in physiology. Am. J. Physiol. Regul. Integr. Compar. Physiol. 304, R1053–R1064. 10.1152/ajpregu.00066.2013 23576606 PMC4073891

[B35] Ripperger J. A. Jud C. Albrecht U. (2011). The daily rhythm of mice. FEBS Lett. 585, 1384–1392. 10.1016/j.febslet.2011.02.027 21354419

[B36] Rocha F. A. D. F. Gomes B. D. Silveira L. C. D. L. Martins S. L. Aguiar R. G. de Souza J. M.. (2016). Spectral sensitivity measured with electroretinogram using a constant response method. PLoS ONE. 11, e0147318. 10.1371/journal.pone.0147318 26800521 PMC4723306

[B37] Roper T. J. (1976). Sex differences in circadian wheel running rhythms in the Mongolian gerbil. Physiol. Behav. 17, 549–551. 10.1016/0031-9384(76)90121-9 1013201

[B38] Saito M. Nishimura K. Kato H. (1989). Modifications of circadian cortisol rhythm by cyclic and continuous total enteral nutrition. J. Nutr. Sci. Vitaminol. 35, 639–647. 10.3177/jnsv.35.639 2517510

[B39] Schilling A. Gerum R. C. Krauss P. Metzner C. Tziridis K. Schulze H. (2019). Objective estimation of sensory thresholds based on neurophysiological parameters. Front. Neurosci. 13, 481. 10.3389/fnins.2019.00481 31156368 PMC6532536

[B40] Schilling A. Krauss P. Gerum R. Metzner C. Tziridis K. Schulze H. (2017). A new statistical approach for the evaluation of gap-prepulse inhibition of the acoustic startle reflex (GPIAS) for tinnitus assessment. Front. Behav. Neurosci. 11, 198. 10.3389/fnbeh.2017.00198 29093668 PMC5651238

[B41] Schilling A. Krauss P. Hannemann R. Schulze H. Tziridis K. (2020). Reduktion der Tinnituslautstärke. HNO. 69, 891–898. 10.1007/s00106-020-00963-5 33185745 PMC8545742

[B42] Searchfield G. Jerram C. Wise K. Raymond S. (2007). The impact of hearing loss on tinnitus severity. Austr. New Zealand J. Audiol. 29, 67–76. 10.1375/audi.29.2.6720339684

[B43] Shannon C. E. (1948). A mathematical theory of communication. Bell Syst. Techn. J. 27, 379–423. 10.1002/j.1538-7305.1948.tb01338.x30854411

[B44] Sheppard A. Liu X. Alkharabsheh A. Chen G.-D. Salvi R. (2019). Intermittent low-level noise causes negative neural gain in the inferior colliculus. Neuroscience. 407, 135–145. 10.1016/j.neuroscience.2018.11.013 30458217

[B45] Siepka S. M. Takahashi J. S. (2005). Methods to record circadian rhythm wheel running activity in mice. Methods Enzymol. 393, 230–239. 10.1016/S0076-6879(05)93008-5 15817291 PMC3770725

[B46] Stephan F. K. (1983). Circadian rhythms in the rat, Constant darkness, entrainment to T cycles and to skeleton photoperiods. Physiol. Behav. 30, 451–462. 10.1016/0031-9384(83)90152-X 6683413

[B47] Turner J. G. (2007). Behavioral measures of tinnitus in laboratory animals. Prog. Brain Res. 166, 147–156. 10.1016/S0079-6123(07)66013-0 17956779

[B48] Turner J. G. Brozoski T. J. Bauer C. A. Parrish J. L. Myers K. Hughes L. F.. (2006). Gap detection deficits in rats with tinnitus, a potential novel screening tool. Behav. Neurosci. 120, 188–195. 10.1037/0735-7044.120.1.188 16492129

[B49] Tziridis K. Ahlf S. Jeschke M. Happel M. F. Ohl F. W. Schulze H. (2015). Noise trauma induced neural plasticity throughout the auditory system of mongolian gerbils, differences between tinnitus developing and non-developing animals. Front. Neurol. 6, 22. 10.3389/fneur.2015.00022 25713557 PMC4322711

[B50] Tziridis K. Forster J. Buchheidt-Dorfler I. Krauss P. Schilling A. Wendler O.. (2021). Tinnitus development is associated with synaptopathy of inner hair cells in Mongolian gerbils. Eur. J. Neurosci. 54, 4768–4780. 10.1111/ejn.15334 34061412

[B51] Tziridis K. Korn S. Ahlf S. Schulze H. (2014). Protective effects of Ginkgo biloba extract EGb 761 against noise trauma-induced hearing loss and tinnitus development. Neural. Plast. 2014, 427298. 10.1155/2014/427298 25028612 PMC4083883

[B52] Valentinuzzi V. S. Scarbrough K. Takahashi J. S. Turek F. W. (1997). Effects of aging on the circadian rhythm of wheel-running activity in C57BL/6 mice. Am. J. Physiol. 273, R1957–R1964. 10.1152/ajpregu.1997.273.6.R1957 9435649

[B53] Zeng F.-G. Fu Q.-J. Morse R. (2000). Human hearing enhanced by noise. Brain Res. 869, 251–255. 10.1016/S0006-8993(00)02475-6 10865084

