# Supplementary material for: Circadian Sensitivity of Noise Trauma-Induced Hearing Loss and Tinnitus in Mongolian Gerbils
Source: Front Neurosci. 2022 Jun 3;16:830703. doi: 10.3389/fnins.2022.830703 (PMC9204100; doi:10.3389/fnins.2022.830703)
Supplement: Supplementary file 1 [file Image_1.pdf]

Supplementary Figures

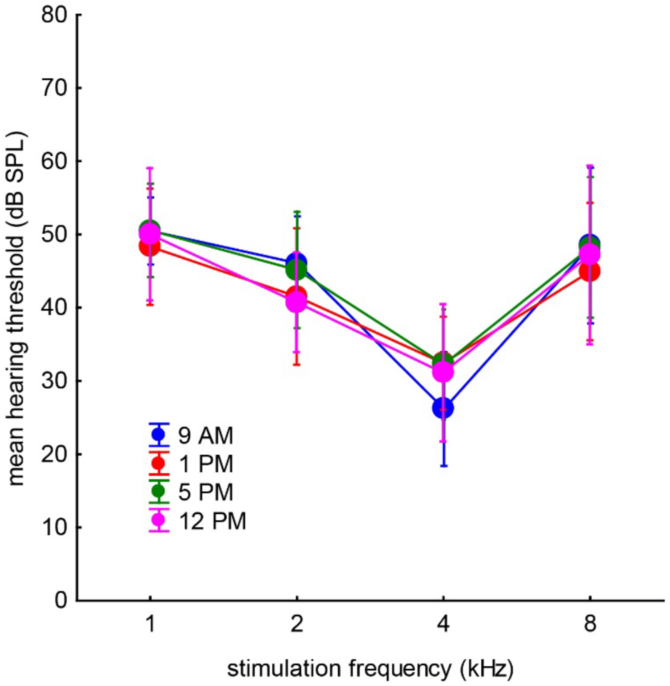

Supplementary Figure 1: Mean hearing threshold of healthy animals before receiving the acoustic trauma. Measurements were always performed at 10 AM (ZT4) on a day before the acoustic trauma. Whiskers depict 95% confidence intervals of the groups.

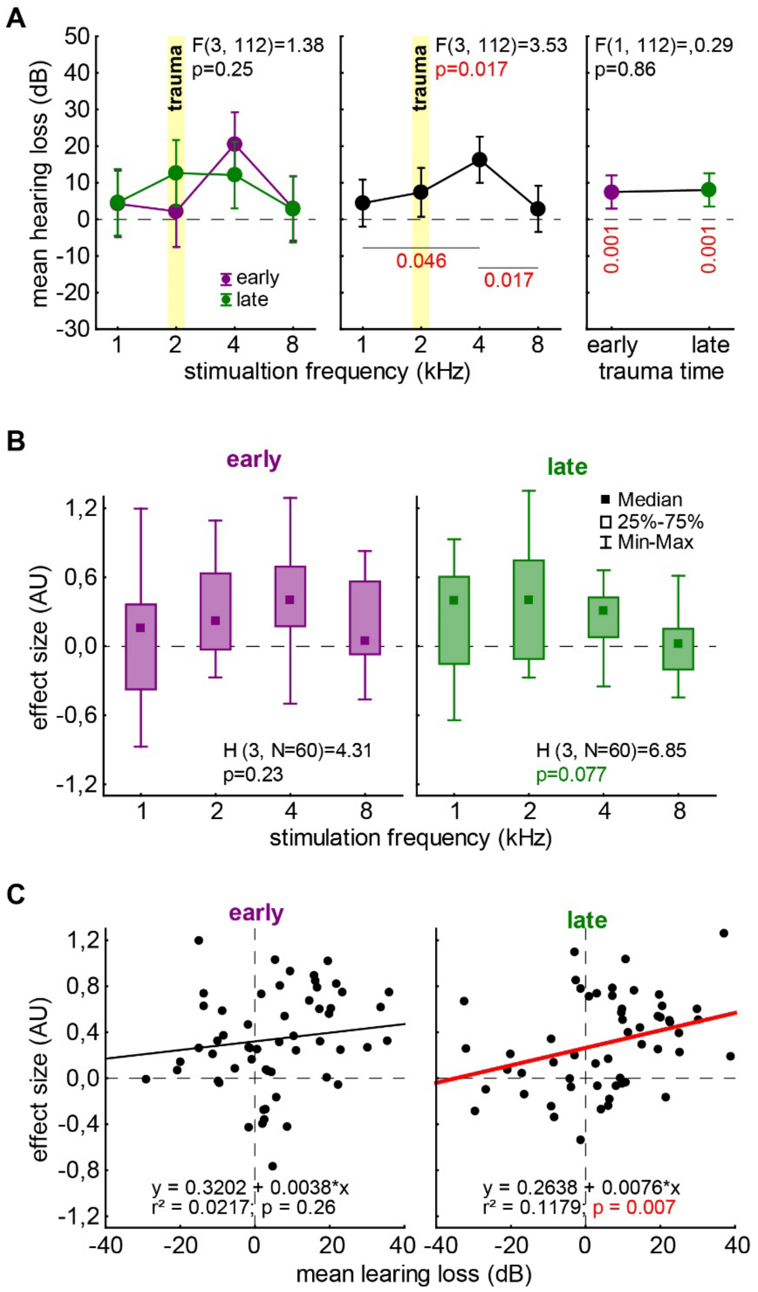

Supplementary Figure 2: **A** 2-factorial ANOVA of mean hearing loss with factors stimulation frequency and trauma time, where trauma time is grouped in “early” (magenta, 9 AM and 1 PM) and “late” (dark green, 5 PM and 12 PM) times of the day. The p values below the lines in the center panel give the significant Tukey post-hoc tests and the perpendicular p values in the right panel give the single sample t-tests. **B** Kruskal-Wallis ANOVAs (H-statistics) of the effect size of the PPI change post vs. pre trauma for the early and late trauma animal groups. **C** Multiple linear regressions for effect size and mean binaural hearing loss for the two trauma time groups.
